# Sustained Reduction of Cerebellar Activity in Experimental Epilepsy

**DOI:** 10.1155/2015/718591

**Published:** 2015-08-31

**Authors:** Kim Rijkers, Véronique M. P. Moers-Hornikx, Roelof J. Hemmes, Marlien W. Aalbers, Yasin Temel, Johan S. H. Vles, Govert Hoogland

**Affiliations:** ^1^Department of Neuroscience, Maastricht University Medical Center, P.O. Box 5800, 6202 AZ Maastricht, Netherlands; ^2^Department of Neurosurgery, Maastricht University Medical Center, P.O. Box 5800, 6202 AZ Maastricht, Netherlands; ^3^School for Mental Health and Neuroscience, Maastricht University, P.O. Box 616, 6200 MD Maastricht, Netherlands; ^4^Department of Neurology, Maastricht University Medical Center, P.O. Box 5800, 6202 AZ Maastricht, Netherlands

## Abstract

Clinical and experimental evidence suggests a role for the cerebellum in seizure control, while no data are available on cerebellar activity between seizures. We hypothesized that interictal regional activity of the deep cerebellar nuclei is reduced in epilepsy and tested this in an animal model by using ΔFosB and cytochrome oxidase (COX) (immuno)histochemistry. The expression of these two markers of neuronal activity was analysed in the dentate nucleus (DN), interpositus nucleus (IN), and fastigial nucleus (FN) of the cerebellum of fully amygdala kindled rats that were sacrificed 48 hours after their last seizure. The DN and FN of kindled rats exhibited 25 to 29% less ΔFosB immunopositive cells than their respective counterpart in sham controls (*P* < 0.05). COX expression in the DN and FN of kindled animals was reduced by 32 to 33% compared to respective control values (*P* < 0.05). These results indicate that an epileptogenic state is characterized by decreased activity of deep cerebellar nuclei, especially the DN and FN. Possible consequences may include a decreased activation of the thalamus, contributing to further seizure spread. Restoration of FN activity by low frequency electrical stimulation is suggested as a possible treatment option in chronic epilepsy.

## 1. Introduction

The cerebellum plays a crucial role in the coordination and control of motor behaviour and cognitive processing [1]. Studies in epilepsy patients and animals models of epilepsy have demonstrated that the cerebellum takes part in the epileptogenic network as well. First of all, case reports have illustrated that the cerebellum can harbour the epileptic focus [2–4]. Furthermore, SPECT and fMRI studies in epilepsy patients have revealed that cerebellar blood flow and blood oxygen level-dependent (BOLD) signal, respectively, increase during secondary generalisation of a partial seizure [5–11]. In rats, cerebellar glucose utilization increases upon administration of proconvulsant drugs [12].

At the cellular level, it has been hypothesized that this ictally increased activity reflects increased GABAergic Purkinje cell firing [13]. Purkinje cells provide the purely inhibitory output of the cerebellar cortex [14]. These inhibitory projections modify the output of the cerebellum through the deep cerebellar nuclei, the dentate nucleus (DN), interpositus nucleus (IN), and fastigial nuclei (FN), which in turn project to the thalamus. This way, the cerebellum regulates activity of neurons elsewhere in the brain.

Thus, seizures are associated with increased activity of the Purkinje cells. Since these cells inhibit the deep cerebellar nuclei, it is likely that seizures are associated with decreased activity of these nuclei. It is not clear whether interictally these cerebellar nuclei are less active as well. The aim of the current study was therefore to assess the regional distribution of interictal neuronal activity in the deep cerebellar nuclei. To this end, we immunohistochemically analyzed cerebellar neuronal activity in fully amygdala kindled rats by evaluating the expression of ΔFosB and cytochrome C oxidase (COX, a.k.a. complex IV), in the DN, IN, and FN.

## 2. Materials and Methods

### 2.1. Animals

Experimental procedures were carried out in 12-week-old male Sprague-Dawley rats (Harlan, Horst, Netherlands). Animals were housed under controlled conditions, a 12-hour light/dark cycle (lights on 7 a.m.), background noise, and food and water* ad libitum*. Adequate measures were taken to minimize pain and discomfort. All experimental procedures were approved by the animal ethics committee of Maastricht University and complied with governmental legislation.

### 2.2. Amygdala Kindling

#### 2.2.1. Electrode Placement

For amygdala kindling, rats were stereotactically implanted with an electrode in the left basolateral amygdala. Perioperative pain was minimized by administering 0.1 mL buprenorphine hydrochloride (Temgesic, Schering-Plough Inc., Amstelveen, Netherlands) subcutaneously 30 minutes before surgery. Next, rats received general isoflurane anesthesia (5% for induction and 2.5% for maintenance throughout the surgical procedure) and were fixed in a stereotactic frame (Dual Manipulator Lab Standard Stereotact, Stoelting Inc., Wood Dale, USA). The implanted electrodes were designed and manufactured by the Department of Instrument Development, Engineering and Evaluation of Maastricht University in collaboration with Professor Dr. Y. Temel (Maastricht University Medical Center, Maastricht, Netherlands). An electrode set consisted of a bipolar stimulating/recording electrode that was implanted in the left basolateral amygdala (coordinates relative to bregma: 2.5 mm posteriorly, 4.8 mm laterally, and 9.6 mm ventrally [15]) and three monopolar stainless steel electrodes that were implanted in the cortex at 1 mm depth. One cortical electrode was used for EEG, one for reference, and one for ground. Connectors for the kindling/EEG electrodes were fixed on the skull using dental acrylic.

#### 2.2.2. Afterdischarge Threshold

Ten days after surgery the prekindling afterdischarge threshold (pre-KADT) was assessed in all rats by stimulating the amygdala with a series of pulses of increasing intensity starting at 10 *μ*A (2 seconds, 50 Hz, 0.2 ms block pulse). Stimuli were delivered through a WPI Accupulser A310 connected to a WPI Stimulus Isolation Unit A360 (World Precision Instruments, Sarasota, FL, USA). EEG registrations from the amygdala and cortical electrodes were recorded from one minute before the kindling stimulus until the end of the behavioural seizure using a Vanguard system (Vanguard Systems, Cleveland Clinics Foundation, Cleveland, USA). Recordings were made with a sample frequency of 200 Hz, a frequency band of 0.5–70 Hz, and a 50 Hz notch filter. The afterdischarge threshold was defined as the stimulus amplitude necessary to elicit a two-second discharge with high frequency and high voltage. One day after the last kindling stimulus, a postkindling afterdischarge threshold (post-KADT) was assessed. The difference between pre-KADT and post-KADT (ΔADT) was calculated by subtracting the respective values and was considered an indirect measure of excitability.

#### 2.2.3. Amygdala Kindling

Amygdala kindling started two weeks after surgery by administering two stimulations per day with an interstimulus interval of at least six hours. Each stimulus lasted two seconds and consisted of a 50 Hz, 400 *μ*A, 0.2 ms block pulse. Seizure severity was assessed based on Racine's scale [16] (i.e., stage 1: freezing; stage 2: head nodding and blinking; stage 3: unilateral forelimb clonus; stage 4: bilateral forelimb clonus and rearing; stage 5: falling). Fully kindled animals were defined as animals that displayed a stage five seizure upon each of five consecutive amygdala stimulations. These fully kindled animals were subsequently stimulated once per day for two more weeks. Kindling rate was defined as the number of stimuli needed to reach the fully kindled state. Sham animals underwent the same surgical procedure and afterdischarge assessments but were not subjected to daily stimulation.

### 2.3. Histological Processing

Two days after the last seizures, that is, 24 h after the assessment of the post-KADT, the rats received an overdose of pentobarbital (75 mg Nembutal/kg bodyweight), followed by perfusion with Tyrode buffer (in mM: 136.9 NaCl, 2.7 KCl, 0.2 MgCl_2_, 11.9 NaHCO_3_, 0.3 NaH_2_PO_4_, and 5.0 glucose, equilibrated with 5% CO_2_/95% O_2_) and then with 4% paraformaldehyde dissolved in 0.1 M phosphate buffer, pH 7.6. The brains were removed, postfixed in the same fixative (4°C, 24 hours), rapidly frozen using CO_2_, and stored at −80°C until sections were cut.

Coronal 10 *μ*m serial sections from the level of the central canal fourth ventricle junction (13.6 mm posteriorly to bregma according to Paxinos [15]) caudally to the inferior colliculus (located 9.6 mm posteriorly to bregma) were cut on a cryostat, mounted on gelatin-coated glass slides, and stored at −20°C until they were processed for (immuno)histochemistry. To minimize staining-to-staining variations, staining experiments always included an equal amount of control and kindled samples.

#### 2.3.1. ΔFosB Immunohistochemistry

Sections were rinsed in Tris-buffered saline containing 0.3% Triton (TBS-T) and then incubated (4°C, 48 hours) with polyclonal rabbit-anti-mouse for B antibody (sc-48, Santa Cruz Biotechnology Inc., Santa Cruz, CA, USA) diluted 1 : 250 in TBS-T containing 0.5% bovine serum albumin. Next, sections were rinsed in TBS-T and incubated (room temperature, overnight) with donkey-anti-rabbit biotinylated antibody (Jackson ImmunoResearch Laboratories Inc., West Grove, PA, USA) diluted 1 : 400 in TBS-T containing 0.5% bovine serum albumin. Sections were then rinsed with TBS-T, incubated for two hours in avidin/biotin diluted 1 : 800 (Vectastain ABC-kit, Vector Laboratories Inc., Burlingame, CA, USA), and visualised by 3.3′-diaminobenzidine (DAB) containing NiCl_2_. Finally, sections were dehydrated and coverslipped.

#### 2.3.2. COX Histology

COX was histochemically detected as described previously [17, 18]. Briefly, sections were air-dried and subsequently incubated (37°C, four hours) in the dark in a nickel enhanced 0.1 M 4-(2-hydroxyethyl)-1-piperazineethanesulfonic acid (HEPES) solution (1% NiCl_2_, pH 7.4) containing 0.0224% cytochrome C oxidase, 0.115% DAB, and 4.5% sucrose. The reaction was stopped by transferring the sections to an ice cold pH neutral buffered 4% paraformaldehyde medium for ten minutes. Finally, sections were dehydrated and coverslipped.

#### 2.3.3. Quantification of Staining

The DN, IN, and FN were photographed in all sections at 4x magnification using an Olympus AX70 bright-field microscope with an Olympus DP70 camera (analySIS Imaging System, Münster, Germany). A mean of 10 sections per animal (range 4–16) for the ΔfosB staining and 16 sections per animal (range 10–19) for the COX staining were analysed. The deep cerebellar nuclei were easily identifiable due to the contrast between nuclei and surrounding white matter, allowing delineation using the freehand drawing function in ImageJ (version 1.43). Tissue artifacts were excluded from analysis.

Cells showing ΔfosB immunoreactivity in the nucleus, cytoplasm, or both were counted by an observer that was blinded for the treatment and expressed as number of immunopositive cells per square millimeter. In total, 129 sections from nine kindled animals and 72 sections from six sham animals were analysed.

Cytochrome oxidase staining was analysed by optical density (OD) using ImageJ. For each nucleus, the OD value was corrected for background staining by subtracting the OD of the adjacent white matter, resulting in negative values as ImageJ applies an inverted grey value scale. In total, 92 stained sections from six kindled animals and 101 sections from six sham animals were analysed. Due to technical problems during the staining sections from three kindled rats could not be analysed.

### 2.4. Statistical Analysis

A Shapiro-Wilk test was performed as test for normal distribution of data. Part of the results were not normally distributed; therefore, the data were analysed using a Mann-Whitney *U* test. Data are expressed as mean ± standard error of the mean (SEM). *P* < 0.05 was considered statistically significant.

## 3. Results

There were no significant differences in ΔFosB immunopositive cell densities or in COX grey values between left and right cerebellar nuclei in shams or in kindled animals; therefore values from both hemispheres were pooled.

### 3.1. ΔFosB

**Figure 1:**
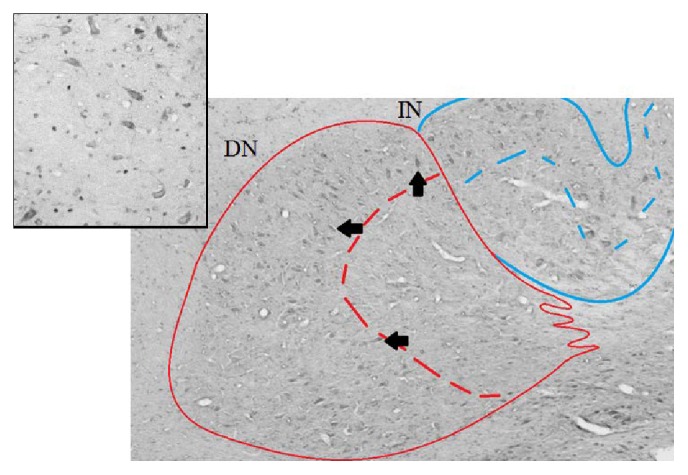
Photomicrograph (10x magnification) showing the distribution of ΔFosB immunoreactive cells in the lateral deep cerebellar nuclei. The cytoplasmic staining of ΔFosB is seen in relatively large cells (arrows) that are almost exclusively localized in the outer regions of the nuclei. These cells are seen in the deep cerebellar nuclei of both sham and amygdala kindled rats. The inset is a 40x magnification showing the cytoplasmic staining in more detail. DN: dentate nucleus; IN: interpositus nucleus.

ΔFosB staining was not confined to the cell nucleus, as would be expected from a nuclear staining, but was seen in the cytoplasm as well (see Figure 1). This “ΔFosB-like immunoreactivity” was almost exclusively found in the most lateral regions of the deep cerebellar nuclei and was observed in the DN in particular.

**Figure 2:**
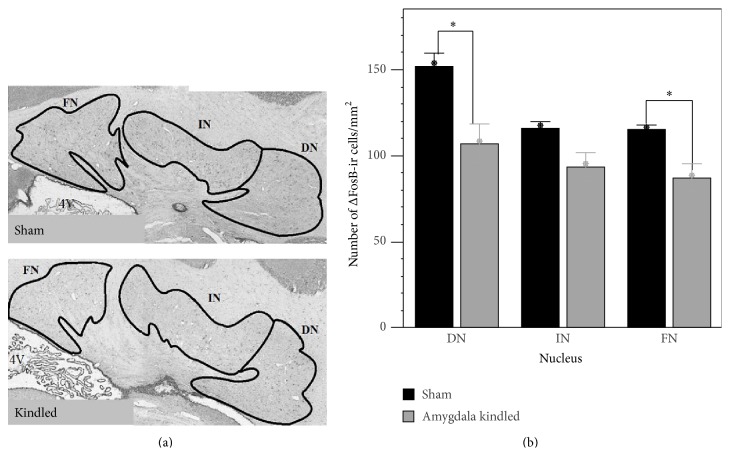
(a) Representative photomicrographs (4x magnification) showing ΔFosB immunoreactivity in the deep cerebellar nuclei of a sham and a fully amygdala kindled rat. Note the overall reduction in ΔFosB immunoreactivity in the kindled rat, suggesting a general decrease in neuronal activity. Coronal sections were taken at bregma level −11.30. 4V: fourth ventricle; DN: dentate nucleus; FN: fastigial nucleus; IN: interpositus nucleus. (b) Number of ΔFosB immunoreactive (ir) cells/mm^2^ in the deep cerebellar nuclei. Data represent mean ± SEM of 72 sections from 6 sham animals and 129 sections from 9 kindled animals. ^∗^ *P* < 0.05.

Density of ΔFosB immunoreactive (ΔFosB-ir) cells was lower in the DN and FN of kindled animals than in shams (DN 108.5 ± 12.4 versus 152.1 ± 7.7, *P* < 0.05, a reduction of 29%; FN 87.2 ± 7.8 versus 115.5 ± 2.2, *P* < 0.05, a reduction of 25%); see Figure 2. In the IN, the density of ΔFosB-ir cells was also lower in kindled than in sham animals; however, this difference was not statistically significant (IN 91.3 ± 9.1 versus 116.2 ± 3.5; *P* = 0.088).

### 3.2. COX

**Figure 3:**
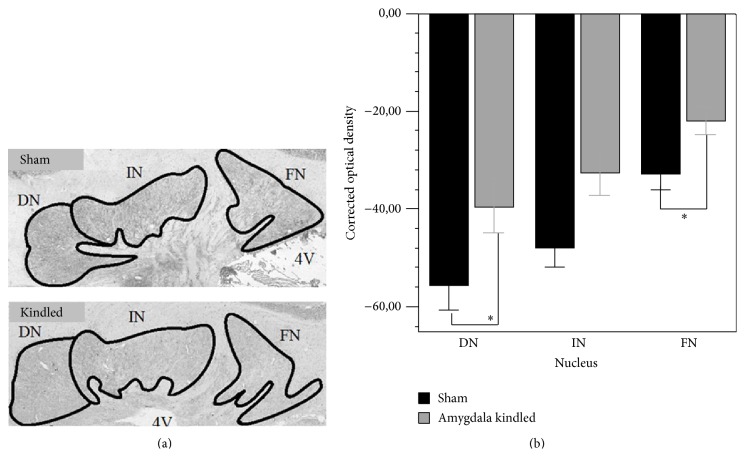
(a) Representative photomicrographs (4x magnification) showing COX activity in the deep cerebellar nuclei of a sham and a fully amygdala kindled rat. Note the overall reduction in COX activity in the kindled rat, suggesting a general decrease in neuronal activity. Coronal sections were taken at bregma level −11.30. 4V: fourth ventricle; DN: dentate nucleus; IN: interpositus nucleus; FN: fastigial nucleus. (b) Corrected optical density of COX activity in the deep cerebellar nuclei. The optical density was corrected for background staining by subtracting the optical density of the adjacent white matter, leading to a negative value. Data represent mean ± SEM of 101 sections from 6 sham animals and 92 sections from 6 kindled animals. ^∗^ *P* < 0.05.

Kindled animals expressed significantly less COX than shams in both DN and FN (DN: −38.2 ± 6.3 versus −55.8 ± 5.2, *P* < 0.05, a reduction of 32%; FN: −22.0 ± 2.8 versus −32.9 ± 3.3, *P* < 0.05, a reduction of 33%; see Figure 3). In the IN, COX expression was also lower in kindled animals than in shams, but this difference was not statistically significant (−32.5 ± 4.7 versus −47.9 ± 4.2; *P* = 0.065).

## 4. Discussion

In this study, we assessed the level of neuronal activity in deep cerebellar nuclei during the interictal phase of fully kindled rats. The observed reductions in activity markers ΔFosB and COX in DN and FN suggest that a decreased seizure threshold is accompanied by reduced activity of the deep cerebellar nuclei.

### 4.1. Methodological Considerations

All experiments were carried out in fully kindled animals that were sacrificed 48 hours after their last kindled seizure. This means that we investigated interictal changes in a chronic epilepsy model. Sham controls did not suffer from seizures. This may have led to a difference in overall locomotor activity between kindled animals and sham controls. Data on interictal cerebellar activity in humans are scarce and are indicative of hypometabolism on PET [19] and SPECT [20]. Epileptic dogs did not show any change in cerebellar metabolism interictally [21]. These imaging studies mainly estimate cerebellar cortical activity, while the deep cerebellar nuclei are not evaluated this way. It is therefore not possible to compare our data with in vivo imaging data.

Some areas of the brain are more involved in generalized tonic-clonic seizures than others [9, 22–24]. The degree of involvement can be immunohistochemically determined by markers for immediately early genes, such as c-fos, FosB, and ΔFosB. C-Fos and FosB peak at two and six hours after a stimulus, respectively. ΔFosB is a highly stable FosB isoform that persists in the brain for several weeks after an initial stimulus [25]. Since the kindling protocol involves repetitive stimuli with interstimulus intervals of more than six hours, we chose to determine the ΔFosB immunopositive cell density as a marker of neuronal activation. To the best of our knowledge, no data are available in literature on basal cerebellar ΔFosB levels nor on cerebellar ΔFosB expression in epilepsy. Our results in sham rats reveal that basal expression of ΔFosB is present in all deep cerebellar nuclei and that, among the deep nuclei, the DN contains the highest density of ΔFosB immunopositive cells. The DN receives input from the more lateral parts of the cerebellar cortex while IN and FN receive input from the vermian cortex (for a review see Voogd [26]). Our data suggest that, in shams, the vermian cortex has a stronger inhibitory effect on its target nuclei than the cortex of the cerebellar hemispheres.

Furthermore, Fos immunoreactivity has been described to occur in three subcellular expression patterns, that is, solely nuclear, solely cytoplasmic, and both [27]. We found cells with nuclear ΔFosB immunoreactivity as well as cells with cytoplasmic staining. This phenomenon has been described for the hippocampus, dentate gyrus, and amygdala by others [28] and appears to be present in the deep cerebellar nuclei as well. The implication of this finding is unclear as cytoplasmic Fos staining has been described weeks to months after a stimulus and has been related to damaged or dying cells [29], while others suggest that this type of expression is related to a mechanism counteracting excitotoxicity [27, 30].

COX is a large transmembrane protein located in mitochondria. It transforms redox energy from the oxidative respiratory chain into a proton-motive force across the mitochondrial membrane. In this process, it receives an electron from four cytochrome c proteins and donates these electrons to free oxygen molecules, thereby converting them to water [31, 32]. Under physiological conditions, COX is involved in the cellular energy supply, while, in the process of seizure-induced neurodegeneration, it plays a role in the activation of proapoptotic pathways [12, 33–37]. Here, we used COX as a marker of oxidative metabolism. COX activity has been shown in the rat cerebellum previously (e.g., by using spectrophotometry and immunoblot [38]), but cerebellar COX expression has not been evaluated before using COX histology. On the other hand, COX histology has been used before to quantify the degree of cerebral oxidative metabolism after electrical stimulation in the rat [18].

### 4.2. Epilepsy Affects Activity of the Deep Cerebellar Nuclei

In kindled animals, the FN and DN showed the largest reduction in ΔFosB expression. Our COX data indicate as well that FN and DN are more affected by kindling than the IN (see Figure 3).

Apparently, amygdala kindling decreases FN and DN activity more than it reduces the activity of IN. This may be the result of direct antidromic effects of electrical stimulation of the amygdala, bearing in mind the possible anatomical connections between the FN, the DN, and the amygdala [38]. These connections have been suggested to exist based on several findings. First, changes in amygdala-related behaviour have been observed following low frequency stimulation of the FN [39] or lesioning of the deep cerebellar nuclei [40]. Second, electrophysiological studies have shown that both low [39] and higher frequency stimulation [38] of the FN can evoke bilateral responses in hippocampus and amygdala. Third, lesioning the FN leads to a bilateral degeneration of synaptic terminals in the amygdala [38]. Alternatively, the cerebellar nuclei may have been affected by the generalized seizures characterizing this model. In line with this notion, cerebellar changes have also been found in epilepsy patients suffering from generalized seizures [41, 42]. This suggestion is furthermore supported by the fact that the generalized seizure lasted up to ten minutes, while the amygdala stimulation lasted only two seconds, and by our observation that there were no left/right differences in staining patterns. Thus, decreased activity of the deep cerebellar nuclei may have been secondary to multiple generalized seizures.

Generalized seizures are associated with increased activity of the cerebellar cortex [5–11]. This cortex has purely inhibitory output [14] provided by GABAergic Purkinje cells [13] and projecting to the deep cerebellar nuclei. Decreased activity of the deep cerebellar nuclei could therefore result from ictally increased Purkinje cell firing. Experimental activation of the Purkinje fibers by electrical stimulation of the cerebellar cortex has anticonvulsive effects in animals [43, 44] and has been investigated as an anticonvulsive therapy in humans as well, with varying results [45–48]. To treat a condition that is associated with increased activity of the cerebellar cortex by activating this same cerebellar cortex may sound contradictory. The expected anticonvulsive effect can best be explained by its subsequent modulation of the deep cerebellar nuclei. Modulation of the deep cerebellar nuclei using electrical stimulation has been performed in an attempt to treat epilepsy patients [49–53], with varying results.

Output of the FN is putatively glutamatergic [53] and decreased activity of the FN may lead to a decreased activation of its most important target structure, the thalamus. This may facilitate further spread of seizures, since the thalamus has been shown to play an important role in seizure control [54–56]. Hence, the FN is of particular interest with regard to the treatment of epilepsy. It has been shown previously that both low frequency stimulation of this nucleus and stimulation of the vermian cortex (which projects to the FN) are anticonvulsive [44, 52]. In contrast, GABA agonist injections into the FN significantly decrease seizure threshold [57]. Furthermore, complete destruction of the FN is proconvulsive, while partial destruction inhibits seizures [58]. In addition, FN lesions abolish the anticonvulsive effects of vermis stimulation [59]. On the other hand, low frequency stimulation of the DN does not affect seizure activity [60] and GABAergic injections into the DN do not affect seizures [57]. This may mean that the role of the DN in seizure control is less prominent.

## 5. Conclusion

The results from this study suggest that the deep cerebellar nuclei are hypoactive in amygdala kindled rats. This hypoactivity was most pronounced in the FN. Functionally located between limbic system, seizure generator, and thalamus, seizure propagator, the FN is an interesting target for epilepsy treatment.

## Abbreviations

BOLD: Blood flow and blood oxygen level-dependent
DN: Dentate nucleus of the cerebellum
IN: Interpositus nucleus of the cerebellum
FN: Fastigial nucleus of the cerebellum
COX: Cytochrome C oxidase
pre-KADT: Prekindling afterdischarge threshold
post-KADT: Postkindling afterdischarge threshold
OD: Optical density.
